# Preparation of Denitrification Materials with Nickel Slag for Nitric Oxide Decomposition in Cement Kilns

**DOI:** 10.3390/ma16175859

**Published:** 2023-08-27

**Authors:** Yanling Gan, Wenjing Dai, Pingli Huang, Boge Zhang, Suping Cui

**Affiliations:** 1 School of Environmental Science and Engineering, Sun Yat-sen University, No. 135, Xingang Xi Road, Guangzhou 510275, China; daiwj7@mail.sysu.edu.cn (W.D.); 13610595675@163.com (P.H.); zhangbg3@mail2.sysu.edu.cn (B.Z.); 2 Guangdong-Hongkong-Macau Joint Laboratory of Collaborative Innovation for Environmental Quality, No. 855 Xingye Avenue East, Guangzhou 511443, China; 3 College of Materials Science and Engineering, Beijing University of Technology, 100 Pingleyuan, Chaoyang District, Beijing 100124, China

**Keywords:** nickel slag, NO decomposition, cement kilns, divalent iron, trivalent iron

## Abstract

NOx emission from the cement industry have received much attention. In order to reduce the NOx emission in cement kilns, nickel slag was used to prepare the non-ammonia denitrification material, and a denitrification mechanism was proposed in this study. The results showed that the denitrification material prepared at pH 7 exhibited the best denitrification performance. At low temperature, the highest denitrification performance was achieved between 200 and 300 °C with a NO decomposition rate of approximately 40%. Then, the NO decomposition rate increased as the temperature increased, reaching over 95% above 700 °C. The physicochemical characteristics showed that the material had the highest specific surface area and the highest relative Fe content, which benefited the denitrification performance. The divalent iron of the denitrification material was considered the active site for the reaction, and trivalent iron was not conducive to denitrification performance at a low temperature range. After the denitrification reaction, the Fe^3+^/Fe^2+^ increased from 0.89 to 1.31. The proposed denitrification mechanism was the redox process between divalent iron and trivalent iron. This study not only recycles industrial waste to reduce solid waste pollution but also efficiently removes nitrogen oxides from cement kilns without ammonia.

## 1. Introduction

Nitrogen oxides (NOx) emission from fossil fuel combustion threatens human health and the environment by photochemical smog, acid rain, ozone layer depletion, etc. Nowadays, after thermal power plants and vehicle exhaust emissions, NOx from cement kilns have become the third largest emission, accounting for approximately one-tenth of the total emissions [1]. As a result, much attention has focused on reducing the nitrogen oxides emitted by cement kilns in recent years. In cement kilns, NOx are primarily formed in the rotary kiln and the precalciner, and more than 95% of the nitrogen oxides produced is NO [2]. At present, low NOx combustion (LNC), selective non-catalytic reduction (SNCR), and selective catalytic reduction (SCR) have been widely applied to remove the NOx emitted from cement industrial processes [3,4,5]. The efficiency of LNC is not sufficient to meet the air emission standards set by the cement industry. A large amount of dust in the tail gas of cement kilns can easily lead to the deactivation of the SCR catalyst. Catalyst deactivation and high cost limit the development of SCR technology. In the application of SNCR to reduce NOx emission, the excessive addition of ammonia can result in severe ammonia leakage and subsequent pollution [6,7]. Direct catalytic NO decomposition does not require reducing agents, such as ammonia or hydrocarbons, and the product is environmentally friendly N_2_ and O_2_; therefore, it is considered the most ideal method for NOx removal. The Gibbs free energy for the direct decomposition of NO has a large negative value in thermodynamics; however, this is a kinetically slow reaction due to a significant activation energy (364 kJ/mol) to overcome. Thus, various catalytic materials have been studied, such as noble metals [8], transition metal ion-exchanged zeolites [9,10], perovskites [11,12], and rare earth oxides [13].

In order to effectively reduce NOx emission in cement kilns, a kind of denitrification material was injected into the preheater with cement raw meal without ammonia or other reducing agents and removed nitrogen oxides during the process of entering the cement rotary kiln. This method demands that the addition of the denitrification materials does not affect the structure and performance of the cement; therefore, the chemical composition of the denitrification materials must be similar to that of the cement. Gan [14] prepared Cu-Al/SiO_2_ porous material, and it was injected directly into the preheater with cement raw meal to decompose NO directly. However, this material is too expensive to use in cement kilns due to the use of chemical reagents as raw materials. Thus, it is important to synthesize an efficient and economical porous material for NOx removal.

Industrial wastes, such as coal fly ash, coal gasification slag, nickel slag, steel slag, etc., have the potential to produce porous materials that are low cost, easy to source, and suitable for industrial production [15]. Alkali-activated nickel slag cementitious materials (ANC) were made using three alkali activators [16]. Wu et al. used nickel slag and metakaolin as the primary raw materials to prepare the porous ceramics material with low bulk density and high porosity [17]. Moreover, industrial waste contains large amounts of transition metals that can be used for denitrification. It has good application prospects not only in terms of energy saving and environmental protection but also in terms of low price and high efficiency. For example, due to the abundance of catalytic active substances, such as rare earth and iron in rare earth tailings, rare earth tailings-based denitrification catalysts have been prepared to reduce NOx emission via NH_3_-SCR [18,19]. The denitrification efficiency of coal gasification slag was systematically examined under different temperatures and concentrations of oxygen [20]. Nickel slag is produced during the smelting of metallic nickel and is a granulated slag of molten material that consists mainly of FeO-SiO_2_; in addition, it is one of the largest industrial waste slag emissions. The main components of nickel slag are silica, alumina, calcium oxide, iron oxide, and so on, which are similar to those of cement. Therefore, in order to produce efficient denitrification materials for cement kilns using industrial waste residues, nickel slag was used in this study to prepare the non-ammonia denitrification material with the chemical precipitation method. The denitrification performance and characterization of the prepared materials were investigated, and the mechanism of the denitrification material was explored. This study not only recycles and utilizes industrial waste nickel slag to reduce solid waste pollution but also efficiently removes nitrogen oxides from cement kilns without the addition of reducing agents such as ammonia.

## 2. Materials and Methods

### 2.1. Preparation of Denitrification Materials

**Figure 1:**
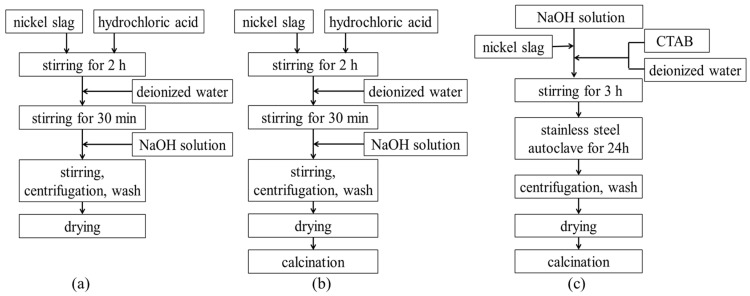
The flow chart of different processes of preparing denitrification materials. (**a**) chemical precipitation method; (**b**) chemical precipitation calcination method; (**c**) hydrothermal method.

Figure 1 shows the flow chart of the different processes for the preparation of denitrification materials ((a) chemical precipitation method; (b) chemical precipitation calcination method; (c) hydrothermal method). The process of preparing denitrification materials with the chemical precipitation method was as follows. A total of 10 g of nickel slag was quickly mixed with 60 mL of hydrochloric acid (3 mol/L). The mixture was stirred sufficiently for 2 h. A total of 50 mL of deionized water was added to the beaker, and the mixture was stirred for another 30 min. NaOH solution (2 mol/L) was slowly added to the mixed emulsion. The pH of the mixed emulsion was adjusted to 3, 7, or 11, respectively. In order to make the mixed emulsion more uniform, the mixture was continuously stirred for 30 min. The mixture was then centrifuged (at a speed of 3000 r/min for 5 min) and washed three times. The precipitate was dried at 80 °C for 24 h. The process of chemical precipitation calcination was to add a calcination process after the chemical precipitation process. The material obtained from chemical precipitation was calcined at 500 °C for 2 h in air.

The process of preparing denitrification materials using the hydrothermal method was as follows. Cetyltrimethylammonium bromide (CTAB) was dissolved in 5 mL of deionized water. A total of 10 g of nickel slag powder and CTAB were rapidly mixed with NaOH solution. The molar composition of the gel mixture that was obtained was SiO_2_: 0.1CTAB: 0.3NaOH: 45H_2_O. During the process, the pH of the solution was adjusted to 10~12. The mixture was sufficiently stirred for 3 h and then transferred into a stainless-steel autoclave and maintained at 100 °C for 24 h. The precipitate was filtered and dried at 80 °C for 24 h, and then the product was calcined at 500 °C for 2 h in air.

### 2.2. Denitrification Performance Test

**Figure 2:**
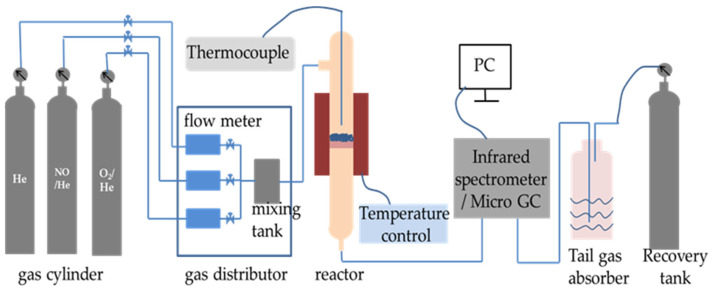
The experimental setup for decomposition of NO.

The denitrification material was injected into the preheater and then gradually fed into the precalciner and rotary kiln with cement raw meal. The reaction temperature was gradually increased to over 1000 °C. In order to imitate the denitrification reactions in the chemical laboratory, the NO decomposition reaction was carried out in a quartz fixed-bed reactor on a laboratory scale and heated with an electrically controlled heating oven, as shown in Figure 2 [14]. The decomposition reaction of NO was conducted in a quartz tube reactor filled with 2 mL of denitrification material. The thermocouple was positioned at the reactor’s core to measure the temperature of the test. The overall flow rate was 300 mL/min. The conditions for the reactant gases were as follows: 1000 ppm NO and helium (He) balanced gas. The materials and reactant gas were heated using a heating rate of 15 °C/min from ambient temperature to 1000 °C. The concentrations of nitric oxide (NO), nitrogen dioxide (NO_2_), and nitrous oxide (N_2_O) were continuously monitored using a TENSOR 27 Fourier-transform infrared (FT-IR) spectrometer (Bruker, Germany), and the NO decomposition rate was calculated according to the following equation:

NO decomposition rate = ([NO]_in_ − [NO]_out_ − [NO_2_]_out_ − [N_2_O]_out_)/[NO]_in_ × 100% (1)

### 2.3. Characterization

The TriStarII 3020 gas sorption analyzer (Micromeritics, Georgia, GA, USA) was used to determine the pore structure, specific surface area, and pore size distribution of the prepared denitrification materials by performing N_2_ adsorption–desorption curves via BET analysis. Before conducting the BET surface measurements, all samples were thoroughly dried at 90 °C for an hour and then degassed for three hours at 350 °C in a vacuum. X-ray diffraction was used to determine the crystal structures of the prepared porous materials. The X-ray diffraction (XRD) patterns were recorded using a Bruker D8 Advance diffractometer (Karlsruhe, Germany) that was equipped with a Cu–Kα radiation source (λ = 0.15405 nm) and operated at 40 kV and 40 mA. The scanning angle (2θ) was measured from 10° to 60°. The diffraction patterns were utilized to identify the phases of the crystalline materials or crystalline grains in the powder. The surface morphology and structure of the denitrification materials were examined using scanning electron microscope (SEM) analysis (SU8020, Hitachi, Japan). The surface composition and valence state of the catalytic materials were mainly analyzed using X-ray photoelectron spectroscopy (XPS) (Thermo ESCALAB 250XI, Waltham MA, USA). The XPS spectra were calibrated to the reference energy of the carbon 1s nuclear level at 284.5 eV.

## 3. Results and Discussions

### 3.1. Denitrification Performance

**Figure 3:**
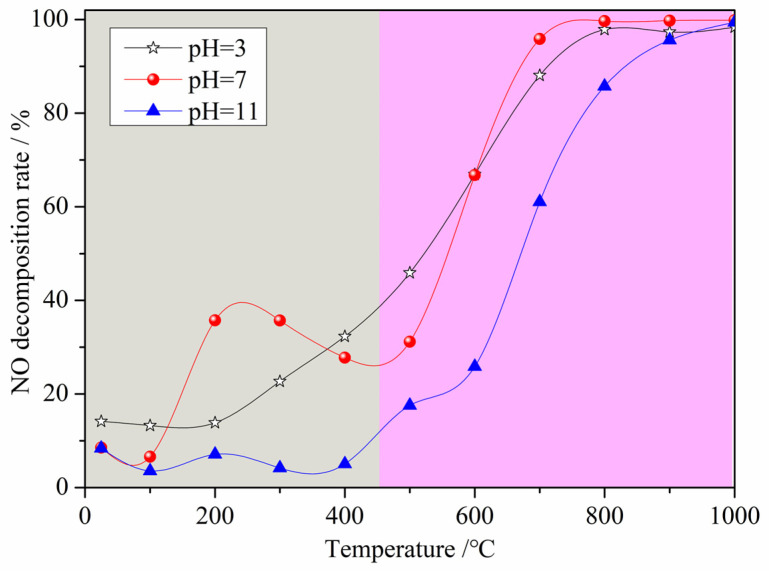
Effect of pH on the NO decomposition rate of the denitrification materials.

Figure 3 displays the NO decomposition rate of the denitrification materials prepared with nickel slag as the raw material with the chemical precipitation method at different pH values. From Figure 3, it can be observed that the changes in the NO decomposition rate of the three denitrification materials with increasing temperature are mainly divided into two trends with a limiting temperature of 450 °C. When the denitrification material was added to the preheater with cement raw material, the temperature gradually increased. When the temperature was below 450 °C, the NO decomposition rate of the three denitrification materials was relatively low, and the denitrification performance of the materials prepared at pH 7 was better than the others. There was an increasing and then decreasing trend in the denitrification performance of the materials prepared at pH 7. The highest denitrification performance was achieved between 200 and 300 °C with a NO decomposition rate of approximately 40%. When the temperature was above 450 °C, the NO decomposition rate of the three denitrification materials increased with increasing reaction temperature. The NO decomposition rate of the material prepared at pH 3 was higher at a temperature between 450 °C and 600 °C. However, when the temperature was above 600 °C, the NO decomposition rate of the denitrification material prepared at pH 7 was still higher, and the NO decomposition rate was higher than 95% at temperatures above 700 °C. Based on the composition of the nickel slag, it is known that the transition metal iron was considered to be the active element for NO decomposition, and the surface oxygen defects of the transition metal oxides were the active sites [21,22,23,24,25]. The active element iron was retained as much as possible by adjusting the pH during the reaction, while the framework elements such as silicon and aluminum were retained, and the elements not conducive to denitrification such as calcium and magnesium were removed with the chemical precipitation method. In the low temperature range, as the denitrification reaction progressed, surface oxygen defects were filled with oxygen atoms generated by NO decomposition. Due to the low temperature, the adsorbed oxygen was not easily desorbed, resulting in a decrease in the number of active sites that, in turn, led to a decrease in the denitrification performance. This is the reason for the trend of first increasing and then decreasing in the low temperature range. As the temperature increased, the oxygen adsorbed on the active sites was gradually easily desorbed, the activity of the active site was restored, and the denitrification performance was improved [26].

The composition of the raw nickel slag and denitrification materials prepared with different pH values was analyzed with X-ray fluorescence spectroscopy (XRF) and X-ray diffraction (XRD). The results of the chemical composition analysis of the raw nickel slag and the prepared denitrification materials are summarized in Table 1. The main chemical compositions of the materials were SiO_2_, MgO, CaO, Fe_x_O, and Al_2_O_3_. During the process of treating the raw nickel slag with the chemical precipitation method, an acid solution was mainly used to leach out metal ions, and an alkaline solution was added to precipitate some of the metal ions. The main purpose of producing denitrification porous materials using nickel slag as a raw material with the chemical precipitation method is to retain framework elements such as silicon and aluminum and active elements such as iron, remove or reduce elements such as calcium and magnesium that are not conducive to denitrification, fully utilize the composition of the nickel slag, and improve the denitrification performance. From Table 1, it can be observed that the content of magnesium and calcium elements decreased, indicating that some magnesium and calcium elements were still present in the liquid phase as ionic species after the addition of the alkali solution and were then washed out, thus achieving the objective of reducing the content of unfavorable elements. Apart from the decrease in iron content in the denitrification material prepared at pH 3, the content of silicon, aluminum, and iron elements increased in the other materials. This was mainly due to the fact that a less alkaline solution was added at pH 3, and the pH was too low to precipitate all the iron elements. The composition of the denitrification material prepared at pH 11 showed little change compared to the nickel slag raw material, mainly due to the addition of too much alkaline solution, which precipitated more magnesium and calcium elements. In contrast, pH 7 can effectively remove calcium and magnesium while retaining silicon, aluminum, and iron. Therefore, the denitrification material prepared at pH 7 had a higher NO removal rate and better denitrification performance than at pH 3 and 11.

**Figure 4:**
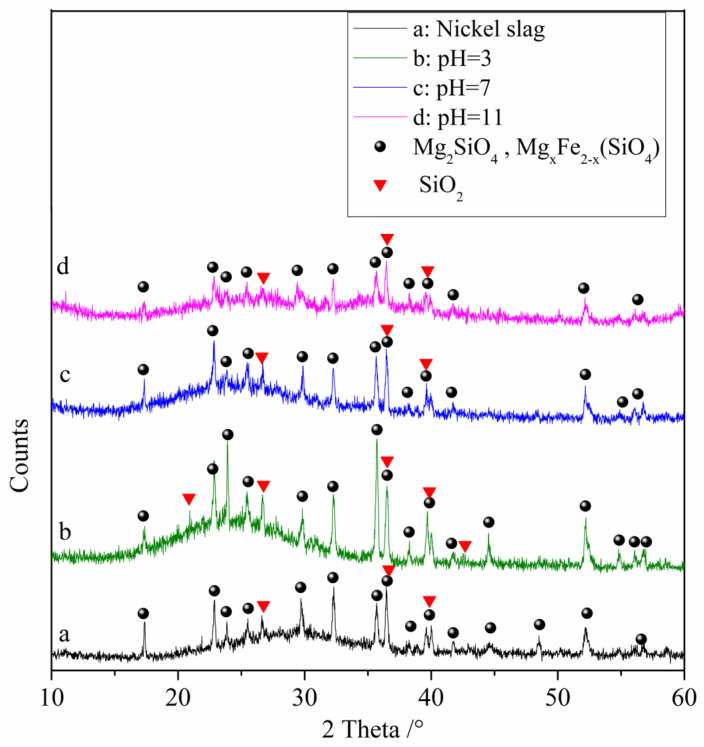
XRD analysis of raw nickel slag and the denitrification materials prepared at different pH values.

The results of the XRD analysis of the raw nickel slag and the denitrification materials prepared at different pH values are shown in Figure 4. From the figure, it can be observed that the diffraction pattern contains large bulges, indicating that nickel slag contains a large amount of amorphous substances, which is determined by the production process of nickel slag. Nickel slag is obtained by rapid cooling after high temperature melting. During rapid cooling, the high temperature glass phase does not have time to crystallize, so it exists in an amorphous form after cooling. The phase analysis showed that the main phases in the raw nickel slag and denitrification materials prepared at three pH values were the amorphous phase Mg_2_SiO_4_, the solid solution Mg_x_Fe_2−x_ (SiO_4_), and the SiO_2_ crystalline phase in which part of the iron replaced magnesium atoms. With increasing pH, the intensity of the crystalline phase peaks gradually decreased, indicating a gradual decrease in the relative content of the crystalline phase. The XRD pattern of the denitrification material at pH 11 showed that some crystalline phases had broadened peaks, forming two amorphous peak bulges, which also indicated that the relative content of the crystalline phase decreased.

**Figure 5:**
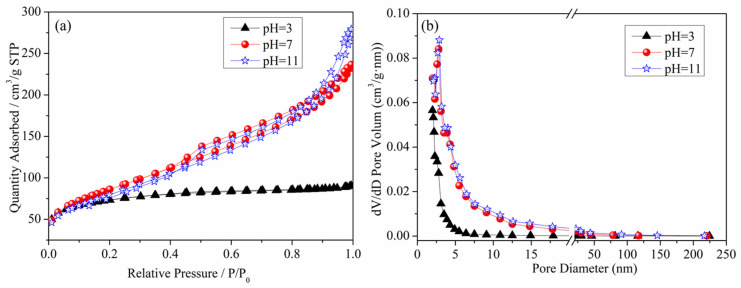
Effect of pH on the N_2_ adsorption–desorption isotherm curve (**a**) and pore size distribution curve (**b**) of the denitrification materials.

Figure 5 presents the N_2_ adsorption–desorption isotherm curve and pore size distribution curve of the denitrification materials. From the figure, it can be observed that the N_2_ adsorption capacity of the denitrification material prepared at pH 3 was significantly lower than that of the denitrification materials prepared at pH 7 and pH 11. This denitrification material had the smallest specific surface area (236.92 m^2^/g), the smallest cumulative pore volume, and the smallest pore size, as shown in Table 2. The denitrification materials prepared at pH 3 had H4 hysteresis loops, indicating that this material was a solid material with narrow cracks. Compared with the H4-type hysteresis loop of the denitrification material prepared at pH 3, the denitrification materials prepared at pH 7 and pH 11 had H3-type hysteresis loops, had a large nitrogen adsorption capacity at high pressure, were mainly flake granular materials or stacked slit porous materials, and had no obvious adsorption saturation platform of the adsorption–desorption isotherm curve. From the pore size distribution diagram of the denitrification materials (Figure 5b), it can be observed that the pore size distribution of the denitrification material prepared at pH 3 was more concentrated, mainly distributed at 2–10 nm. The corresponding pore volume was smaller than that of the denitrification materials prepared at pH 7 and 11, and the specific surface area was the smallest, indicating that the material prepared at pH 3 had fewer and smaller pores. The pore size of the materials prepared at pH 7 and 11 was mainly distributed between 2 and 50 nm, and the pore volume was relatively large, indicating that the materials contained more mesopores.

Furthermore, based on Table 2, the test results of specific surface area, pore volume, and pore size of the denitrification materials prepared at different pH values indicate that the denitrification material prepared at pH 7 had the highest specific surface area of 308.1921 m^2^/g, cumulative pore volume of 0.3627 cm^3^/g, and average pore size of 5.0769 nm. For denitrification materials, the larger the specific surface area, the larger the contact area with the NO gas during the denitrification reaction. The active elements on the denitrification material are more likely to come into contact with the NO gas, resulting in better denitrification performance. Therefore, the denitrification material prepared at pH 7 had the best denitrification performance.

**Figure 6:**
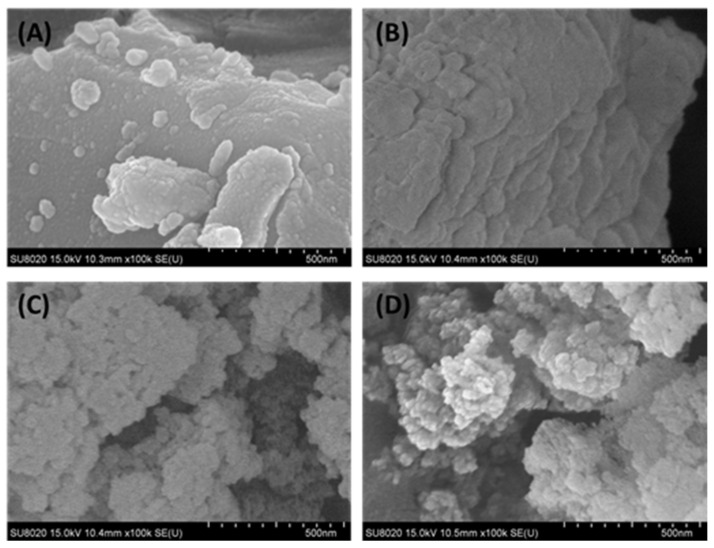
Surface morphology of the raw nickel slag and denitrification materials prepared at different pH values ((**A**): raw nickel slag, (**B**): pH = 3, (**C**): pH = 7, (**D**): pH = 11).

The surface morphology of the raw nickel slag and denitrification materials prepared at different pH values was analyzed with a scanning electron microscope, as shown in Figure 6. From Figure 6B, it can be observed that the denitrification materials prepared at pH 3 were mainly closely distributed in a lamellar structure, and there were many amorphous gels with a compact structure. This is consistent with the BET analysis, which showed that this material had a small specific surface area and a type-IV adsorption–desorption isotherm with an H4-type hysteresis loop. The denitrification materials prepared at pH 7 and 11 were clearly characterized by a loose-particle stacking structure with a relatively large specific surface area (see Figure 6C,D), which was more conducive to the adsorption of NO gas on the surface of the denitrification material and promoted the improvement of denitrification performance.

**Figure 7:**
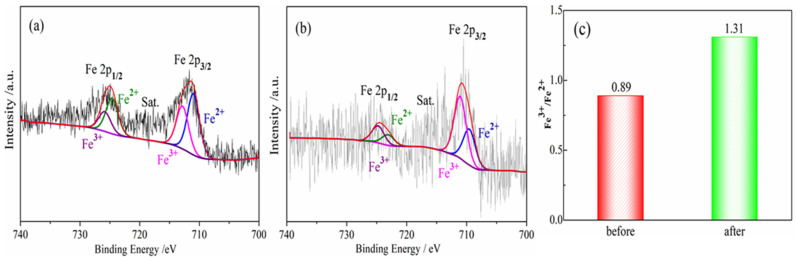
Fe 2p XPS spectra for porous materials prepared by nickel slag before and after denitrification. (**a**) before reaction; (**b**) after reaction; (**c**) Fe^3+^/Fe^2+^ before and after denitrification.

The denitrification material prepared at pH 7 with the best denitrification performance was taken as an example to analyze the Fe 2p XPS spectra of the material before and after denitrification. Figure 7 shows the Fe 2p XPS spectrum of the denitrification materials prepared with nickel slag before and after the reaction. The XPS spectrum of Fe 2p was mainly divided into two peaks: the first peak at 705–720 eV belonging to the Fe 2p 3/2 orbit and the second peak at 720–730 eV belonging to the Fe 2p 1/2 orbit, as presented in Figure 7a,b. The peak position of Fe 2p 3/2 has been investigated, and it has been reported that the values of the peak position of Fe 2p 3/2 are often between 710.6 and 711.2 eV [27,28,29]. According to the peak separation of Avantage v5.52 software, two obvious fitting peaks at 710.3 eV and 712 eV for Fe 2p 3/2 correspond to the binding energy of Fe^2+^(2p 3/2) and Fe^3+^(2p 3/2) [30]. By calculating the atomic content of iron in different valence states, it can be concluded that the atomic ratio of trivalent iron (Fe^3+^) to divalent ferrous iron (Fe^2+^) in the denitrification material prepared at pH 7 was 0.89, while the atomic ratio of trivalent iron (Fe^3+^) to divalent ferrous iron (Fe^2+^) in the product after the denitrification reaction was 1.31, as shown in Figure 6C. After the denitrification reaction, the relative content of trivalent iron increased significantly, indicating that, during the denitrification reaction with NO, the redox reaction of Fe in the denitrification material occurred, an electron was transferred from Fe^2+^, and Fe^3+^ was produced; in addition, the electron was transferred to NO, thereby promoting the decomposition of NO.

### 3.2. The Effect of Fe Valence State in Denitrification Materials

**Figure 8:**
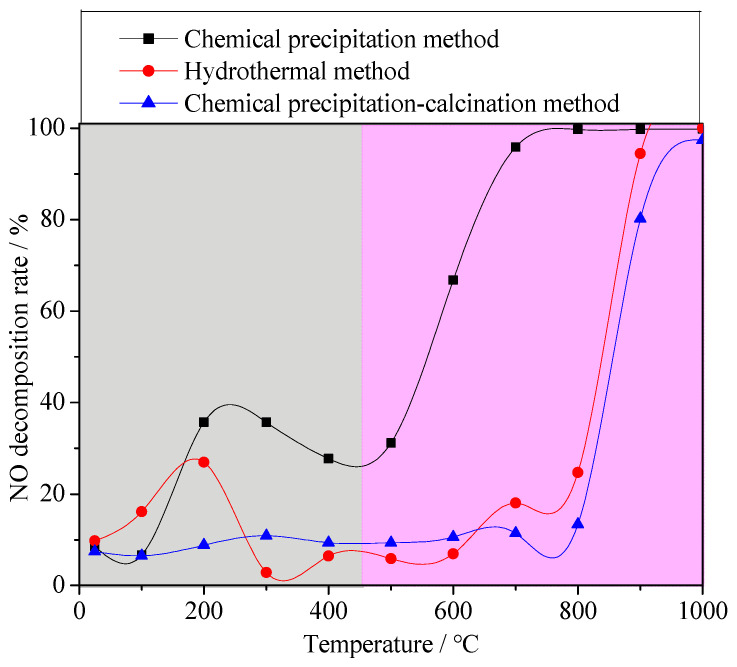
NO decomposition rate of the denitrification materials prepared with different methods.

In order to study the effect of the Fe valence state in materials on denitrification performance, different synthetic methods were used to synthesize the denitrification materials. The denitrification performance analysis of the material prepared with the chemical precipitation method showed that the denitrification material prepared at pH 7 had the highest specific surface area and the best NO decomposition rate. Therefore, with the same chemical ingredients, the denitrification material with the better pore structure was prepared with the hydrothermal method using cetyltrimethylammonium bromide (CTAB) as a template, and their denitrification performance was compared. Due to the calcination process in the hydrothermal synthesis process, calcination in an air atmosphere may affect the valence states of the elements. Another denitrification material was prepared with the chemical precipitation–calcination method, and its NO decomposition rate was tested as a comparative sample. Figure 8 shows the NO decomposition rate of the denitrification materials prepared with different methods. It can be clearly observed from the figure that the NO decomposition rate of the denitrification material prepared with the chemical precipitation method was much better than that of the denitrification material prepared with the hydrothermal method and the chemical precipitation–calcination method.

**Figure 9:**
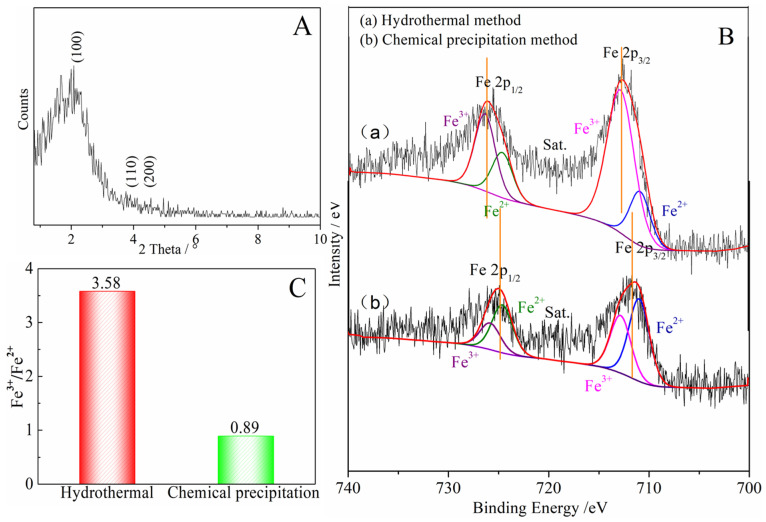
(**A**) Low-angle XRD pattern of the denitrification material prepared with the hydrothermal method; (**B**) Fe 2p XPS spectra for the denitrification materials prepared with different methods; (**C**) Fe^3+^/Fe^2+^ of the denitrification materials prepared with the hydrothermal method and chemical precipitation method.

Compared with the pore structure of the denitrification materials prepared with the chemical precipitation method, the material prepared with the hydrothermal method had a larger specific surface area of 514.0633 m^2^/g and a smaller average pore size of 3.1982 nm, as shown in Table 3. In addition, the X-ray small-angle diffraction result of the denitrification material prepared with the hydrothermal method (see Figure 9A) indicated that the material had the characteristic diffraction peak (100) of MCM-41 at approximately 2° as well as the broadening and weakening peaks (110) and (200), indicating that the denitrification material synthesized with the hydrothermal method had the structural characteristics of MCM-41 mesoporous material with a large specific surface area and a regular pore structure. Although the material prepared with hydrothermal synthesis had a better structure with a large specific surface area and regular pore structure, its denitrification performance was low, indicating that this was affected by the active components (Fe) in the material. Moreover, the difference between the chemical precipitation method and the chemical precipitation–calcination method was whether calcination was carried out after the drying process, but the final denitrification performance was significantly different. Therefore, the change in iron caused by the calcination process was considered to be the main reason for the decrease in denitrification performance.

The Fe 2p XPS spectra of the denitrification materials prepared with different synthetic methods are illustrated in Figure 9B. According to the peak separation and calculation using Avantage v5.52 software, the atomic ratio of trivalent iron (Fe^3+^) to divalent ironion (Fe^2+^) in the denitrification material prepared with the hydrothermal method was 3.58, while the atomic ratio of trivalent iron (Fe^3+^) to ferrous iron (Fe^2+^) in the material prepared with the chemical precipitation method was 0.89, as shown in Figure 9C. The content of ferric iron (Fe^3+^) in the material prepared with the hydrothermal synthesis method increased sharply, indicating that the calcination of the hydrothermal synthesis method converted Fe^2+^ into Fe^3+^. From the comparison between (a) and (b) in Figure 9B, it can be observed that, compared with the position of the Fe 2p peak of the material prepared with the chemical precipitation method, the positions of the Fe 2p 3/2 and Fe 2p 1/2 peaks of the material prepared with the hydrothermal method moved to the position with higher binding energy, indicating that the iron oxide changed during the hydrothermal synthesis process, causing the transfer of energy and the transformation of some divalent iron into ferric iron. The divalent iron tended to lose electrons to NO, thereby promoting the decomposition of NO, whereas trivalent iron cannot effectively promote the decomposition of NO. However, only at high temperatures, trivalent iron was converted to divalent iron, which promoted the decomposition of NO. Therefore, although the specific surface area of the material prepared with the hydrothermal method was large, and the pore structure was more conducive to the adsorption of active elements (Fe) and NO gas, its denitrification performance was significantly lower than that of the material produced with the chemical precipitation method.

### 3.3. Denitrification Mechanism

**Figure 10:**
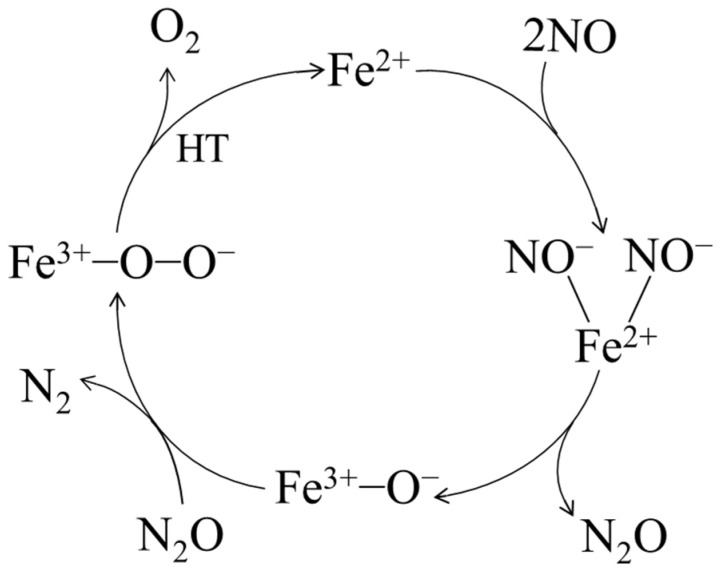
Proposed denitrification mechanism of the material prepared with nickel slag.

The results of denitrification performance and physicochemical characteristics showed that the denitrification material prepared with the chemical precipitation method with nickel slag at pH 7 had the best NO decomposition performance because it had the highest specific surface area and the highest relative Fe content. The Fe in nickel slag was proposed as the active metal element [22,31]. Furthermore, the denitrification material prepared with the chemical precipitation method had a better denitrification performance than that of the material prepared with the hydrothermal method, indicating that divalent iron was considered the active site for the denitrification reaction [21,32]. After the denitrification reaction, the relative content of trivalent iron increased significantly, indicating that, during the denitrification reaction with NO, the redox reaction of Fe^3+^ in the denitrification material occurred, an electron was transferred from Fe^2+^, and trivalent iron was produced. According to the analysis results and relevant theories [21,22,32,33,34], a proposed catalytic cycle for NO decomposition on the denitrification material prepared with the chemical precipitation method with nickel slag at pH 7 is shown in Figure 10. First, NO was selectively adsorbed on the Fe^2+^ ion, the N-O bond on Fe^2+^ was more likely to be broken than in the gas phase as an electron was provided from the Fe^2+^ ion to a NO molecule, the bond length of NO was increased, and the N-O bond was activated. Second, the dinitrosyl species adsorbed on the Fe^2+^ ion formed the NO^−^-Fe^2+^-NO^−^ species, which was decomposed to Fe^3+^-O^−^ species and an N_2_O intermediate. N_2_O was easily decomposed to N_2_ and adsorbed O, forming the Fe^3+^-O-O^-^ species. Finally, Fe^3+^-O-O^−^ species was decomposed at high temperature to produce Fe^2+^ and O_2_.

## 4. Conclusions

Nickel slag was used to prepare the non-ammonia denitrification material to remove NO in cement kilns, and a denitrification mechanism was proposed in this study. The results showed that the denitrification material prepared at pH 7 exhibited the best denitrification performance. At a low temperature, the highest denitrification performance was achieved between 200 and 300 °C with a NO decomposition rate of approximately 40%. Then, the NO decomposition rate increased as the temperature increased, reaching over 95% above 700 °C. The physicochemical characteristics showed that the material had the highest specific surface area and the highest relative Fe content, which benefited the denitrification performance. The denitrification performance of the materials synthesized with different synthetic methods showed that the material prepared with the chemical precipitation method had the best NO decomposition rate, which indicated that the divalent iron of the denitrification material was considered the active site for the denitrification reaction, and trivalent iron was not conducive to denitrification performance at a low temperature range. After the denitrification reaction, the Fe^3+^/Fe^2+^ increased from 0.89 to 1.31. The proposed denitrification mechanism was that, during the denitrification reaction with NO, the redox reaction of Fe^2+^ in the denitrification material occurred, an electron was transferred from Fe^2+^, and trivalent iron was produced. Finally, Fe^3+^ was decomposed at high temperature to produce Fe^2+^. This study not only recycled and utilized industrial waste nickel slag to reduce solid waste pollution but also efficiently removed nitrogen oxides from cement kilns without the addition of reducing agents such as ammonia. Considering the gas atmosphere inside the cement kilns, the presence of oxygen and sulfur oxide in the kiln may affect the denitrification performance. Therefore, further research can focus on the effect of the oxygen atmosphere on denitrification and how these materials can be used for desulphurization and denitrification reactions simultaneously.

## Figures and Tables

**Table 1:** Main chemical composition of raw nickel slag and denitrification materials prepared at different pH/wt%.

--	SiO_2_	MgO	CaO	Fe_x_O	Al_2_O_3_
Raw nickel slag	49.57	22.50	15.23	6.18	4.84
pH = 3	77.13	7.62	3.13	2.54	6.68
pH = 7	70.13	7.74	3.19	8.29	6.67
pH = 11	51.12	21.68	11.28	6.54	5.03

**Table 2:** Effect of pH on the specific surface area and pore structure of the denitrification materials.

pH	Specific Surface Area (m^2^/g)	Cumulative Pore Volume (cm^3^/g)	Average Pore Diameter (nm)
pH = 3	236.9211	0.0645	3.1267
pH = 7	308.1921	0.3627	5.0769
pH = 11	289.6233	0.4564	6.1077

**Table 3:** Specific surface area and pore structure of the denitrification materials prepared with different synthetic methods.

Synthetic Method	Specific Surface Area (m^2^/g)	Cumulative Pore Volume (cm^3^/g)	Average Pore Diameter (nm)
chemical precipitation method	308.1921	0.3627	5.0769
hydrothermal method	514.0633	0.443028	3.1982

## Data Availability

The authors confirm that the data supporting the findings of this study are available within the article. Data will be provided upon request.
